# A case report: A patient rescued by VA-ECMO after cardiac arrest triggered by trigeminocardiac reflex after nasal surgery

**DOI:** 10.1097/MD.0000000000035226

**Published:** 2023-09-29

**Authors:** Xu Zhang, Bin Sun, Chen Pac-Soo, Daqing Ma, Liwei Wang

**Affiliations:** a Department of Anaesthesiology, Xuzhou Central Hospital, Xuzhou, China; b Division of Anaesthetics, Pain Medicine and Intensive Care, Department of Surgery and Cancer, Faculty of Medicine, Imperial College London, Chelsea and Westminster Hospital, London, UK; c Department of Anesthesiology, Wycombe General Hospital, High Wycombe, Buckinghamshire, UK.

**Keywords:** cardiac arrest, case report, endoscopic nasal surgery, extracorporal cardiopulmonary resuscitation, trigeminocardiac reflex

## Abstract

**Rationale:**

Cardiac arrest (CA) caused by trigeminocardiac reflex (TCR) after endoscopic nasal surgery is rare. Hence, when a patient suffers from TCR induced CA in the recovery room, most doctors may not be able to find the cause in a short time, and standard cardiopulmonary resuscitation and resuscitation measures may not be effective. Providing circulatory assistance through venous-arterial extracorporeal membrane oxygenation (VA-ECMO) can help healthcare providers gain time to identify the etiology and initiate symptom-specific treatment.

**Patient concerns:**

We report a rare case of CA after endoscopic nasal surgery treated with VA-ECMO.

**Diagnoses:**

We excluded myocardial infarction, pulmonary embolism, allergies, hypoxia, and electrolyte abnormalities based on the relevant examination results. Following a multidisciplinary consultation, clinical manifestation and a review of previous literature, we reasoned that the CA was due to TCR.

**Interventions:**

VA-ECMO was established to resuscitate the patient successfully during effective cardiopulmonary resuscitation.

**Outcomes:**

ECMO was successfully evacuated a period of 190 minutes of therapy. The patient was discharged home on day 8.

**Lessons:**

TCR is notable during endoscopic nasal surgery. Our case indicates that CA in operating room is worth prolonged CCPR. The ideal time for ECPR implementation should not be limited within 20 minutes after CCPR.

## 1. Introduction

Trigeminocardiac reflex (TCR) is a brainstem reflex of parasympathetic origin following stimulation of the sensory branches of the trigeminal nerve, causing a sudden onset dysrhythmias resulting in severe electrocardiographic abnormalities, haemodynamic instability, and other autonomic disturbances leading to apnea and gastric hypermobility.^[1]^ TCR can be initiated following stimulation of the anterior and posterior ethmoidal nerves during nasal surgery.^[2]^ The incidence of TCR among endoscopic nasal surgery was reported to be 4.15% but TCR induced cardiac arrest (CA) was very rare.^[3]^

Herein, we report a case of CA induced by TCR not responding to standard resuscitation protocol. The patient was successfully resuscitated after a period of support with veno-arterial extracorporal membrane oxygenation (VA-ECMO). The patient was understanding of the sudden CA and subsequent treatment with VA-ECMO. He gave written consent for this report to be published.

## 2. Case report

A 74-years-old-male patient (height 170 cm, weight 59 kg) was admitted for an elective surgical resection of a nasal mass under general anaesthsia. He had nasal congestion for 2 years and physical examination revealed a nasal mass. He had coronary heart disease for more than 10 years and was not taking any standard cardiac medication. He denied suffering from hypertension, diabetes and other chronic diseases. Systematic clinical examination and laboratory investigations, including a 12 lead ECG and echocardiogram, were normal. The patient was classified, at preoperative assessment, as an ASA II and a New York Heart Association grade II for level of fitness.

The patient arrived in the operating theater at 12h45. A 20 G cannula was inserted into the left radial artery, under aseptic condition, after local anaesthsia with lidocaine, for haemodynamic monitoring. Before induction of general anaesthsia, the ECG showed a sinus rhythm with a heart rate (HR) of 66 beats per minute (bpm), the arterial blood pressure (ABP) was 145/79 mm Hg, and the arterial saturation, measured with a pulse oximeter, was 97% with the patient breathing air. Blood gas analysis was within normal range.

Anaesthsia was induced with midazolam 2 mg, etomidate 10 mg, sufentanil 30 μg, atracurium maleate 12 mg and maintained with propofol infusion at 3 to 5 mg kg^-1 hour-1^ and remifentanil at 0.2 to 0.3 μg kg^-1 minute-1^. His trachea was intubated and his lungs were ventilated. At the end of surgery, which lasted 65 minutes, the nasal cavity was packed with a sponge to reduce bleeding. Both anaesthsia and surgery were smooth and uneventful. Blood gas analysis was repeated during surgery and all readings were normal. General anaesthtic was discontinued and the patient was transferred to the post-anaesthtic care unit at 14h35.

In the post-anaesthtic care unit, after the patient regained consciousness and was able to obey verbal commands to open eyes and hold hands with strong force and his vital signs (SpO_2_ 100%, ABP 130/70 mm Hg, HR 62 bpm) were stable, the endotracheal tube was removed at 14h39. The patient was able to breath oxygen easily through a face mask, and no complaints was reported by the recovery nurse. He suddenly developed severe bradycardia with a HR of 40 bpm and an associated reduction of ABP to 91/47 mm Hg. Atropine 0.5 mg, dopamine 1 mg and norepinephrine 8 μg were promptly administered intravenously. Unfortunately, the patient’s condition did not improve following these treatments; he developed a third-degree atrio-ventricular block with a ventricular rate of 31 bpm and the ABP dropped further to 54/32 mm Hg at 14h41. The patient lost consciousness at that point and this prompted us to intubate the trachea and ventilate the lungs mechanically with 100% oxygen. Epinephrine 0.2 mg bolus was administered intravenously followed by an intravenous infusion of 0.2 μg kg^-1 minute-1^ to correct the haemodynamic instability. The patient developed CA at 14h43. Cardiopulmonary resuscitation was implemented immediately, epinephrine 1 mg and methylprednisolone 1000 mg were administered intravenously. Myocardial infarction, pulmonary embolism, allergic shock and electrolyte abnormalities were excluded from consideration based on the patient’s medical history, blood gas analysis, electrocardiogram, myocardial enzymes and echocardiography. It is very likely that the CA was caused by the pack in the nasal cavity which triggered the TCR. The sponge was, therefore, removed from the nasal cavity at 14h46 but, unfortunately, the CA persisted.

**Figure 1.**
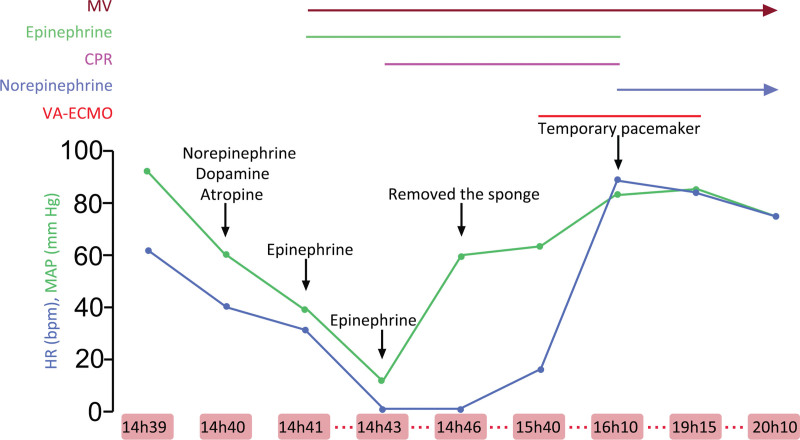
Treatment timeline. CPR = cardiopulmonary resuscitation, HR = heart rate, MAP = mean arterial pressure, MV = mechanical ventilation, VA-ECMO = Veno-arterial extracorporal membrane oxygenation.

At 15h20, the resuscitation team decided to establish a VA-ECMO to improve the success rate of recovery. At 15h40, VA-ECMO was established via the right femoral artery and vein. Soon afterwards, there was return of spontaneous circulation and the patient regained haemodynamic stability with a return of sinus rhythm of about 88 bpm, the ABP was around 122/61 mm Hg, and the patient maintained good oxygenation with an SpO_2_ of 100% when ventilated with an inspired oxygen concentration of 60%. Norepinephrine was intravenously infused at a rate of 0.1 to 0.2 μg kg^-1 minute-1^, while the patient was receiving VA-ECMO support. He was sedated with midazolam 4 mg, paralyzed with cisatracurium besylate 10 mg and he received analgesia with sufentanil 15 μg administered intravenously. Considering the previous third-degree atrio-ventricular block, temporary pacemaker implantation was performed via the left femoral vein. After a period of 190 minutes of VA-ECMO therapy, the patient remained haemodynamically stable, requiring minimal norepinephrine support at 0.03 μg kg^-1 minute-1^; the latter was discontinued. A timeline of the treatments is presented in Figure 1.

The patient was transferred to the intensive care unit for further care at 20h10 and he was then transferred to the general surgical ward the following day. He maintained sinus rhythm with gradually reducing the pacing rate. After adequate evaluation of laboratory inspections and holter electrocardiogram, a cardiologist withdrew the temporary pacemaker 2 days after surgery. He was discharged home 8 days after surgery and he remained well in community with a normal neurological function and good physical activity 3 years after surgery.

## 3. Discussion

CA occurring in patients undergoing scheduled noncardiac surgery during the intraoperative period is very rare with an incidence between 0.8 to 4.3 per 10,000 cases.^[4]^ The common causes include hypoxia, cardiac disease, massive blood loss, anaphylactic reaction and adverse neurological reflexes. We excluded myocardial infarction, pulmonary embolism, allergies, hypoxia, and electrolyte abnormalities based on the relevant examination results. Following a multidisciplinary consultation and a review of previous literature, we reasoned that the CA was very likely due to TCR.

TCR is a well described brain stem reflex characterized by bradycardia and in severe cases, asystole, hypotension, apnea, and enhanced gastrointestinal peristalsis following stimulation of the afferent branches of the trigeminal nerves.^[1]^ Stimulation of the maxillary nerve, the anterior and posterior ethmoidal nerves, during endoscopic nasal surgery, cause afferent signals to travel up to the trigeminal sensory nucleus in the 4th ventricle via the Gasserian ganglion.^[5]^

In our case, the sponge in the nasal passages may have triggered the TCR and caused the asystole. However, this was not reversed by removing the sponge and by the administration of atropine 0.5 mg. It could be argued that a higher dose of anticholinergic should have been administered to block the muscarinic receptors at the sino-atrial node. For example, in a previous study, atropine up to 1.2 mg was required to stop TCR during surgery in the cerebello-pontine angle.^[6]^ Furthermore, in another study during cranial surgery, high doses of atropine had failed to prevent TCR induced bradycardia from occurring.^[7]^

The patient suffered from coronary artery disease and this most likely contributed to the persistence of the asystole in spite of the appropriate action taken to reverse the TCR. Standard cardiopulmonary resuscitation lasted for about 60 minutes before VA-ECMO was initiated and the patient recovered fully. Our case and the case previously reported^[8]^ indicated that medical professionals should not give up resuscitating patients when standard cardiopulmonary resuscitation protocol fail to revive patients but consider adding VA-ECMO to the treatment regime.

Closed thoracic cardiopulmonary bypass, now called ECMO, has been demonstrated to, successfully, resuscitate rabbits who had CA^[9]^ and this has subsequently been shown to be effective in clinical practice too. For example, in a previous study, the authors showed that the use of extracorporal cardiopulmonary circulation during resuscitation resulted in the return of spontaneous circulation in 95% of the subjects compared to 47% in those who had the standard cardiopulmonary resuscitation.^[10]^ Our management of the CA described above is in line with the American Heart Association Guidelines which recommends ECMO in refractory CA.^[11]^ Furthermore, a randomized controlled trial showed that cumulative 6-month survival was significantly better in the group of patients who received early ECMO than in the group who received standard advanced cardiac life support treatment.^[12]^

Our case also presents certain limitations. In scenarios involving procedures with a high likelihood of inducing TCR and in dealing with high-risk patients, we did not react at the earliest opportunity. Furthermore, preventive measures such as anticholinergic medications and local anesthetics were not employed. This could be the cause of the eventual severe CA. In addition, CA caused by TCR was just a conclusion inferred by our team after excluding other factors by relevant data.

Several suggestions have been proposed to help reduce the risk of TCR occurring during surgery, for example, avoiding light anaesthsia, being cautions of beta blockers and calcium channel blocker use and providing adequate pain management peri-operatively for patients undergoing nasal surgery.^[3,13]^ In addition, the administration of NMDA receptor antagonist during induction of general anaesthsia was reported to decrease the risk of oculo-cardiac reflex during strabismus surgery.^[14]^

## 4. Conclusion

We need to take precautions to reduce the risk of TCR occurring during nasal surgery where the operative field is innervated by the trigeminal nerve. If TCR occurs, it should be treated promptly to prevent subsequent serious consequences.

## Acknowledgements

We thank Drs Meiyan Zhou, Yangzi Zhu, Yan Zhang, Congyou Wu, Qian Liu, Li Yan, Kai Wang, and Wenping Ding (Department of Anaesthsiology, Xuzhou Central Hospital) for their assistance during the resuscitation of the patient.

## Author contributions

**Conceptualization:** Xu Zhang, Daqing Ma.

**Data curation:** Xu Zhang, Bin Sun, Chen Pac-Soo, Daqing Ma, Liwei Wang.

**Formal analysis:** Xu Zhang, Bin Sun, Chen Pac-Soo.

**Funding acquisition:** Bin Sun, Liwei Wang.

**Investigation:** Xu Zhang, Bin Sun.

**Methodology:** Bin Sun, Daqing Ma, Liwei Wang.

**Resources:** Bin Sun.

**Supervision:** Daqing Ma, Liwei Wang.

**Validation:** Xu Zhang, Daqing Ma, Liwei Wang.

**Visualization:** Xu Zhang, Bin Sun.

**Writing – original draft:** Xu Zhang, Bin Sun, Liwei Wang.

**Writing – review & editing:** Xu Zhang, Chen Pac-Soo, Daqing Ma, Liwei Wang.
